# Discordant β-Lactam Susceptibility in Clinical *Staphylococcus aureus* Isolates: A Molecular and Phenotypical Exploration to Detect the BORSA/MODSA Isolates in Bogotá, Colombia

**DOI:** 10.3390/microorganisms12122598

**Published:** 2024-12-16

**Authors:** Angie Lorena Fonseca-Fernández, María Alejandra Mancera-García, Aura Lucia Leal-Castro, Chad Leidy, Sandra Rincón, Lina P. Carvajal, Jinnethe Reyes, Adriana Marcela Celis Ramírez

**Affiliations:** 1 Grupo de Investigación Celular y Molecular de Microorganismos Patógenos, Department of Biological Scieces, Universidad de los Andes, Bogotá 111711, Colombia; al.fonseca10@uniande.edu.co (A.L.F.-F.); ma.mancera10@uniandes.edu.co (M.A.M.-G.); 2 Grupo de Investigación en Enfermedades Infecciosas, Faculty of Medicine, Universidad Nacional de Colombia, Bogotá 111321, Colombia; allealc@unal.edu.co; 3 Biophysics Group, Department of Physics, Universidad de los Andes, Bogotá 111711, Colombia; cleidy@uniandes.edu.co; 4 Unidad de Genética y Resistencia Antimicrobiana, Universidad El Bosque, Bogotá 110121, Colombia; rinconsandra@unbosque.edu.co (S.R.); carvajallinap@unbosque.edu.co (L.P.C.); reyesjinnethe@unbosque.edu.co (J.R.)

**Keywords:** MRSA, BORSA/MODSA, *spa*-typing, *pvl*, CzIE, *Staphylococcus aureus*

## Abstract

*Staphylococcus aureus* is a human pathogen responsible for a wide range of diseases, such as skin and soft tissue infections, pneumonia, toxic shock syndrome, and urinary tract infections. Methicillin-resistant *S. aureus* (MRSA) is a well-known pathogen with consistently high mortality rates. Detecting the *mecA* resistance gene and phenotypical profile to β-lactams allows for the differentiation of MRSA from methicillin-susceptible *S. aureus* (MSSA) isolates. In this study, we characterized 57 *S. aureus* clinical isolates for β-lactam susceptibility and *mecA* presence. We classified 52.63% as MRSA and 45.61% as MSSA. However, some isolates evidenced different oxacillin resistance profiles, such as borderline oxacillin-resistant or modified *S. aureus* (BORSA/MODSA). The cefazolin inoculum effect (CzIE) was established for these samples, emphasizing the relevance of these isolates as a source of therapeutic failure. We also performed the detection of the Panton-Valentine Leucocidin virulence genes as well as the *S. aureus spa*-type clonality. As expected, *spa*-types t002 and t008 were the most prevalent clones, demonstrating the success of well-established clones. These findings emphasize the importance of establishing sensitivity profiles, especially in isolates with poor resistance mechanisms, to determine their prevalence and their impact on public health.

## 1. Introduction

*Staphylococcus aureus* is one of the most important human pathogens causing infections worldwide. Penicillin was the first β-lactam antibiotic described and used to treat infections caused by this pathogen. However, penicillin-resistant *S. aureus* cases rapidly increased, prompting the development of methicillin, an alternative semisynthetic β-lactam antibiotic. Nonetheless, methicillin-resistant *S. aureus* (MRSA) strains quickly emerged, showing resistance to all β-lactam and most non-β-lactam antibiotics, including vancomycin, daptomycin, and linezolid [1]. Methicillin resistance is tested by agar diffusion and microdilution conventional methods with β-lactam antibiotics such as oxacillin and cefoxitin [1], as well as the identification of the genes responsible for this antibiotic resistance, such as the *mecA* gene [2] or its homologs, *mecC*, *mecB* and *mecD*, which code for an alternative penicillin-binding protein (PBP2a) that is less affine to β-lactam antibiotics [3]. However, recent findings indicate that the classification between MRSA and MSSA and conventional methods is insufficient [4]. 

Currently, novel β-lactam susceptibility profiles have been identified, such as oxacillin-susceptible *mecA*-positive *S. aureus* (OS-MRSA) strains that are susceptible to β-lactams such as cefoxitin and oxacillin, but still have the *mecA* gene [5]. This leads OS -MRSA isolates to be misidentified as MSSA by conventional methods, which results in inappropriate treatment implementation and increases the possibility of morbidity and mortality in patients [6]. On the other hand, borderline oxacillin-resistant (BORSA) and modified *S. aureus* (MODSA) are isolates with low levels of oxacillin resistance. BORSA/MODSA resistance is not related to *mec* genes. Instead, this is linked to an increase in β-lactamases production (BORSA) or, in some cases, point mutations in native PBPs (MODSA) with oxacillin MICs among 1 and 16 µg/mL [7]. The introduction of these novel antimicrobial profiles highlights the challenges in characterizing the sensitivity profiles of *S. aureus* isolates, hence increasing the challenges associated with managing infections resulting from this pathogenic bacterium. 

On the other hand, MSSA isolates are generally considered easier to treat; however, the high inoculum effect of cefazolin (CzIE) in MSSA is a key factor in therapeutic failure, particularly in invasive infections such as bacteremia. CzIE is characterized by a significant increase in the minimum inhibitory concentration (MIC) of cefazolin (CZ) when the inoculum rises from 1 × 10^5^ CFU/mL to 1 × 10^7^ CFU/mL [8], leading up to cefazolin treatment failure in invasive MSSA infections [9]. Although the inoculum effect has not been directly associated with BORSA or MODSA phenotypes, the beta-lactamase activity plays a major role in the development of CzIE [9], as also in the BORSA phenotype. The mechanism underlying CzIE involves the production of β-lactamases, mediated by extracellular BlaZ enzymes, which vary from types A to D depending on genome sequencing and their affinity to cephalosporins [10]. In this way, the accurate identification of BORSA/MODSA isolates could help to prevent treatment failure and prompt the consideration of alternative antibiotic classes to manage *S. aureus* infections caused by oxacillin-resistant *mec*lacking *S. aureus.*

These findings related to the *S. aureus* susceptibility profiles have underlined the importance of effectively characterizing clinical isolates of this crucial pathogen, and have made it necessary to track the spread of infections by molecular tools. In this regard, the *Panton-Valentine* leukocidin genes (*LukS-PV* and *LukF-PV*) have been studied as primary factors associated with severity in skin and soft tissue infections and have been used to classify the hospital-associated MRSA (HA-MRSA) and community-associated MRSA (CA-MRSA) isolates related to them [11,12,13,14].

Currently, the boundary between this *S. aureus* classification is blurred due to the establishment of genotypic CA-MRSA in hospital settings and the persistence of HA-MRSA in community environments [15]. Recently, livestock-associated methicillin-resistant *S. aureus* (LA-MRSA) has been considered a potential source of human infections. These MRSA strains are primarily linked to the increasing production and consumption of meat, mainly pigs and people with continued exposure to them (veterinaries and caregivers), as well as antibiotic use in animals [16,17]. Although the origin of infection could serve as a proper epidemiological classification, the overlap among strains makes it necessary to establish molecular markers for tracking *S. aureus* infections. These molecular markers have made it possible to establish clonal lineages between clinically important isolates. An example of that is LA-MRSA isolates associated with the clone CC38, also known as CC398, known as an “animal-adapted clade”, which, in addition to the *mecA* gene, usually caries the *tet* gene, confers resistance to tetracycline, and is associated with t011 and t034 *spa*-types [16,18]. *Spa*-typing is a molecular typing method that subtypes *Staphylococcus aureus* isolates by using the sequence of a polymorphic variable number tandem repeat (VNTR) in the protein A (*spa*) [19]. Despite being a single-locus typing technique, *spa* typing provides subtyping resolution comparable to more expensive and time-consuming techniques such as multilocus sequence typing (MLST) and pulsed-field gel electrophoresis (PFGE) [20]. This technique is widely used in hospitals and outbreak settings; it is helpful in studies of pathogen molecular epidemiology, including detecting outbreaks and tracking transmission events [21]. Therefore, this study aims to characterize the molecular and phenotypic profiles of S. aureus clinical isolates as a complementary strategy for identifying and correctly classifying the resistance profiles of this important human pathogen.

## 2. Materials and Methods

### 2.1. Isolates Collection 

A total of 57 clinical isolates provided by the Grupo para el Control de la Resistencia Bacteriana en Bogotá (GREBO) of the Universidad Nacional de Colombia and the Laboratorio de Biofisica of the Universidad de Los Andes, Bogotá, Colombia, were included. These isolates were collected between 2019 and 2020 from body secretions (43.8%) (n = 25); blood cultures (28%) (n = 16); (abscesses) 7% (n = 4); furuncles, bones, breast drains, catheters, and urine (1.75%) (n = 1 each); and other body sites (12.78%) (n = 7). This is a descriptive study considering skin and soft tissue infections (SSTIs), which do not tend to be life-threatening, and therefore could be underestimated.

The isolates were cultured for 24 h at 37 °C on trypticase soy agar (TSA; Scharlau Chemie, Barcelona, Spain) and then stored at −80 °C in Luria–Bertani broth (Difco, Franklin Lakes, NJ, USA) with 15% glycerol until use in subsequent experiments. 

### 2.2. Bacterial Typing 

The DNA extraction was performed following the guidelines of the Zymo Quick DNATM Universal kit (Zymo Research, Orange County, CA, USA) and GenElute^TM^ Bacterial Genomic DNA Kit (Sigma-Aldrich, St. Louis, MO, USA) for PCR gene amplification. Nanodrop 2000/2000c (Thermo Scientific^TM^, Waltham, MA, USA) was used to measure DNA concentration. DNA integrity was confirmed by 0.8% agarose gel electrophoresis at 45 V for 45 min with a 1 Kb ladder (Gentech Biosciences, Envigado Antioquia, Colombia) and HydraGreen^TM^ Safe DNA Dye (ACTGene Inc., Kendall Park, NJ, USA). The extracted DNA was stored at −20 °C until its use.

To identify the *16s rRNA*, *mecA,* and *pvl* genes, as well as *S. aureus spa* clonal typing, we performed fragment PCR amplification with specific primers (Table S1). The molecular amplification was carried out using a 35 µL master mix that contained 1 µL of DNA (150 ng/µL), 17.3 µL of PCR water, 2.5 µL of 10X Buffer Taq, 0.4 µL of dimethyl sulfoxide, 0.2 nM of each dNTP, and 0.2 mM of each primer. The DNA fragments of *16s rRNA* and *spa* genes were confirmed by 1% agarose gel electrophoresis at 45 V for 45 min with a 1 kb ladder (Invitrogen, Carlsbad, CA, USA). *mecA* and *pvl* gene amplification was revealed in a 2% agarose gel by electrophoresis at 80 V for 45 min with a 100 bp ladder (Invitrogen, Carlsbad, CA, USA) and HydraGreen™ Safe DNA Dye (ACTGene Inc., Kendall Park, NJ, USA). The expected band size for *mecA* was 155 bp [22] and 433 bp for *pvl* genes [12].

The *16s rRNA* and *spa* PCR amplification products were sequenced by Sanger capillary electrophoresis (1000 pb ABI 3500 XL) at the GenCore laboratory of the Universidad de Los Andes (https://corefacilities.uniandes.edu.co/gencore/, accessed on 12 April 2020). For *16s rRNA* sequences, the software Geneious Prime 2020.2 (Biomatters, Inc., Newark, NJ, USA) (www.geneious.com, accessed on 12 April 2020) was used to process the obtained sequences. The species-specific isolates were confirmed by BLASTn [23]. Additionally, MEGAX version 10 (Pennsylvania State University, University Park, PA, USA) was used to align the sequences (www.megasoftware.net, accessed on 12 April 2024) and determine the similarity among isolates. Using 1000 bootstraps and the evolutionary model Kimura 2, we built a maximum likelihood phylogenetic tree. As outgroups, we used the *S. saprophyticus* and *S. epidermidis* sequences (NCBI: NR 115607.1 and KR149342.1, respectively). On the other hand, *spa gene* sequences were cleaned, and clones were assigned through BioNumerics software version 8.0 (Applied Maths BVBA, Sint-Martens-Latem, Belgium) (www.applied-maths.com/bionumerics/patches/71/patch, accessed on 20 April 2020). A minimum spanning tree was built to evidence the relationship among *spa*-type isolates and reported MRSA pandemic clones [24].

### 2.3. Antibiotic Susceptibility Testing

The agar diffusion method described in CLSI M100 guidelines was used to test the antibiotic susceptibility of cefoxitin (OXOID), gentamycin (OXOID), ciprofloxacin (OXOID), erythromycin (OXOID), clindamycin (OXOID), and trimethoprim-sulfamethoxazole (OXOID). Additionally, microdilution testing was utilized to assess oxacillin susceptibility testing following CLSI M100 standards [25]. For each antibiotic evaluated, based on the CLSI breakpoints, all isolates were categorized as susceptible, intermediately susceptible, or resistant. The assays were run in triplicate. 

### 2.4. Validation of Discordant Susceptibility Isolates 

To confirm isolates with discordant antibiotic profiles, isolates whose susceptibility profiles to oxacillin and cefoxitin were not the same or the presence or absence of the *mecA* gene could not explain, and specifically isolates first classified as presumptive OS-MRSA (isolates 34, 44, and 1634) and BORSA/MODSA (isolates 7, 28, 37, and 38), we carried out *mecA* detection using a PCR multiplex assay by Rincón and colleagues 2013, which identifies MRSA isolates. Also, the *blaZ* detection was performed using their protocols [26]. Finally, the microdilution susceptibility test for OXA was performed according to CLSI M100 guidelines [25]. These confirmation assays were performed in the Unidad de Genética y Resistencia Antimicrobiana—Universidad del Bosque (UGRA) Bogotá D.C. 

#### 2.4.1. Cefazolin Inoculum Effect (CzIE) Testing 

A microdilution susceptibility test for CZ was performed according to the CLSI M100 guidelines [25] in isolates that presented discordant antibiotic-resistant profiles (presumptive OS—MRSA and BORSA/MODSA). The high inoculum effect was achieved by changing the starting inoculum for the microdilution cefazolin susceptibility test from 1 × 10^5^ UFC/mL to 1 × 10^7^ UFC/mL. The cefazolin high inoculum effect (CzIE) was defined as an MIC value of ≥16 ug/mL at high inoculum with a standard cefazolin MIC ≤ 8 µg/mL. The controls for this susceptibility test were the strains *S. aureus* TX0117 (BlaZ A, CzIE), *S. aureus* ATCC 29213 (BlaZ A, non CzIE), and *S. aureus* ATCC 25923 (non-BlaZ production) [26].

#### 2.4.2. Rapid Colorimetric Test to CzIE

Presumptive OS-MRSA and BORSA/MODSA strains were incubated overnight in brain heart infusion (BHI) agar; then, isolated colonies were taken in a 1 µL calibrated loop and placed in 1 mL BHI broth with an ampicillin disk (150 mg/mL) for the induction and liberation of β-lactamases. The solution was homogenized by vortex for 2 min and incubated for 10 min at 37 °C; this process was carried out twice. Then, it was centrifugated at 5000 rpm for 10 min. Finally, without disturbing the cell pellet, 25 µL of the supernatant was taken and placed in 25 µL of nitrocefin (400 mM). The solution was incubated at room temperature for 15 min and monitored for 2 h. Any color change from yellow to red indicates extracellular β-lactamases liberation, and a positive CzIE result. The control strains for this assay were *S. aureus* TX0117 as a positive control (BlaZ A, CzIE), *S. aureus* ATCC 29213 (*BlaZ* A, non CzIE), and *S. aureus* ATCC 25923 as a negative control (non-BlaZ production) [8].

## 3. Results

This study analyzed and confirmed the 57 clinical isolates of *S. aures* via BLASTn and phylogenetic analysis (Figure S1). Furthermore, antibiotic susceptible profiles were established, demonstrating that 1.75% of the isolates were resistant to gentamicin, 3.5% to ciprofloxacin, 12.28% to clindamycin, and 21% to erythromycin. Moreover, in terms of β-lactam resistance, 54.3% of the isolates were resistant to oxacillin, but only 47.3% were resistant to cefoxitin (Table S2). In addition, the *mecA* gene was only detected in 52.6% of the isolates, and *pvl* virulence genes were found in 49.1% of the studied samples (Table S2). Additionally, we established the *spa* type of those clinical isolates. There were 32 *spa* types found, with clones t008, t002, and t024 being the most common (22.8%, 12.2%, and 10.5%, respectively). Also, the isolates clustered with *spa clones* were closely related to *S. aureus* pandemic clones such as Archaic, Iberian, Pediatric, and NY-Japanese (Figure S2). 

We contrasted *mecA* gene presence with the susceptibility profiles to β-lactam antibiotics (oxacillin and cefoxitin) (Table S2). About 47.4% (n = 27) of the isolates were *mecA* positive and resistant to both β-lactam antibiotics; these were classified as MRSA. In contrast, 40.3% (n = 23) lacked the *mecA* gene and were susceptible to oxacillin and cefoxitin. These were classified as MSSA.

However, 5.2% (n = 3) of the isolates were susceptible to both β-lactam antibiotics tested but had the *mecA* gene, and 7% (n = 4) were resistant to oxacillin despite lacking *mecA*. That led to their classification as discordant isolates within the presumptive BORSA/MODSA and OS-MRSA phenotypes, respectively. However, only two discordant samples, isolates 37 and 38, could be confirmed (Table 1) because those two isolates were categorized as resistant according to their OXA MIC values of 16 µg/mL, despite lacking the *mecA* gene.

Additionally, to infer the possible mechanism behind OXA resistance, *blaZ* gene detection was performed, along with CzIE detection using a rapid chromogenic nitrocefin test and a microdilution cefazolin susceptibility test at a standard and high inoculum. For isolates 7 and 1634, only some of these tests were performed because *mecA* was identified through PCR confirmation, establishing this gene as the mechanism for oxacillin resistance.

These results confirmed concordance between the presence of the *blaZ* gene and the positive chromogenic rapid test for CzIE (Figure S3). This was the case of isolate 37, classified now as the BORSA/MODSA isolate. The CzIE explains cefazolin treatment failure through production of β-lactamases in *S. aureus* with non *mec* genes [8], a resistance mechanism shared with BORSA isolates. In contrast, isolate 38 did not provide evidence for either *blaZ* gene or a positive result for *BlaZ* production and CzIE; however, OXA MIC of 16 µg/mL lacking *mecA* allowed us to classify it as part of the BORSA/MODSA phenotype. On the other hand, isolate 44 was the only one which presented the *blaZ gene* but a negative result for the rapid chromogenic test. Finally, every proven isolate presented CzIE (Table 1).

## 4. Discussion

*Staphylococcus aureus* is a human pathogen capable of adapting to various environments, making it challenging to identify an appropriate treatment to combat its virulence and rapid proliferation. Furthermore, treatment failure is compounded by its increasing resistance to both β-lactam and non-β-lactam antibiotics and the difficulties in determining an accurate antibiotic resistance profile using conventional methods [27]. Therefore, this study aims to describe and assess the phenotypic and molecular antimicrobial resistance profiles of *S. aureus* clinical isolates. It offers a more thorough comprehension of its resistance mechanisms and focuses on the discordant β-lactam susceptibility profiles.

We verified that all clinical isolates included in this study were *S. aureus* and confirmed that they exhibited resistance to both β-lactam and non-β-lactam antibiotics. Interestingly, we determined that some isolates present discordant sensitivity profiles to B-lactam antibiotics (OXA resistance and FOX susceptibility and vice versa), as well as resistance to other antibiotics such as erythromycin (macrolide), clindamycin (lincosamide), gentamicin (aminoglycoside), and ciprofloxacin (fluoroquinolone), (Table S2). Also, the *Panton-Valentine Leucocidin* gene (*pvl*) was found in both MRSA and MSSA isolates. These findings show concordance with previous reports in Colombia, where MRSA rates range from 45 to 50% [11,28], and *pvl* genes are found in both MRSA and MSSA isolates as well [29]. Indeed, we described various *S. aureus spa*-types circulating in the region. However, three well-known *spa* types, t008, t002, and t024, have also been described in Europe, Asia, and America [30]. Furthermore, these predominant *spa* types are associated with MRSA pandemic clones responsible for outbreaks in the UK, Denmark, Portugal, Poland, USA, Argentina, Japan, and Colombia [24], indicating the potential of these isolates to cause reemerging outbreaks.

Moreover, the extraordinary ability of this pathogen to acquire a considerable array of antibiotic resistance factors compromises the efficacy of antibiotic treatment [31]. The therapeutic approach is guided by isolates’ antibiotic susceptibility characterization based on molecular and phenotypical techniques like *mecA* gene detection, broth microdilution, and agar dilution tests [32]. Therefore, a precise susceptibility profile is essential to prevent therapeutic failure. We confirmed that 47% of the isolates were MRSA and 40% were MSSA. However, two BORSA/MODSA isolates were detected (Table 1) because the *mecA* gene was not detected in these isolates by either PCR technique; its OXA MIC was 16 µg/mL according to the microdilution test (Table 1). These results allowed us to classify both as BORSA/MODSA *S. aureus*; however, the resistance mechanism behind their β-lactams resistance needs to be elucidated.

Although BORSA/MODSA isolates are distinguished for presenting 1–8 ug/mL OXA MICs, recent studies have also shown oxacillin MICs with higher values [7], evidencing the increasing resistance to β-lactams even in these barely known phenotypes. Thus, isolate 38 presented a negative result for the *blaZ* gene and CzIE (Table 1, Figure S3). This may indicate that the resistance mechanism to β-lactams could be due to possible modifications in PBP native enzymes, which decrease the affinity of β-lactams [33]. In contrast, isolate 37 presented the *blaZ* gene and BlaZ positive detection through the chromogenic rapid test, which allowed us to consider hyper-production of β-lactamases as the oxacillin resistance mechanism. It is important to highlight that CzIE is mediated by β-lactamase activity, as for the BORSA phenotype. This suggests a potential correlation between these phenotypic features in *S. aureus* lacking *mec*; however, only one BORSA isolate in our study (37 sample) did not allow us to establish a definitive correlation. Additionally, the CzIE implies a high bacterial density (1 × 10^7^ UFC/mL), while the BORSA resistance mechanism occurs at standard (1 × 10^5^ UFC/mL) bacterial inoculum, both through β-lactamase activity.

On the other hand, isolate 44 (first classified as OS-MRSA) presented the *blaZ* gene, but evidenced a negative result to BlaZ detection according to the rapid chromogenic test. This discrepancy could be due to the sensitivity of the rapid test, where nitrocefin has more affinity to BlaZ type A enzymes (92.7%) than the other BlaZ types. In that way, we could hypothesize that this isolate codified to BlaZ type B or C, and the rapid test is significantly less sensitive in detecting these types of β-lactamases (53.3% and 72.3%, respectively) [8].

Recently, some authors have focused on resistance determinants of β-lactams different from the well-known *mec* and *blaZ* genes. Not only modifications in PBP 1–4 native proteins decrease affinity to β-lactams; those in the *pbp4* promoter do as well, enhancing bacterial cell wall crosslinking and wall rigidness [33], mutations in the cation multidrug efflux transporter AcrB [33], and modifications in the cyclic di-AMP phosphodiesterase (GdpP) responsible for cyclic di-AMP cleavage, a regulator of bacterial homeostasis and growth affecting bacterial tolerance to β-lactams [34]. The best approach for studying BORSA/MODSA isolates would be through whole-genome sequencing, focusing on modifications in native *S. aureus* proteins as a new perspective for understanding BORSA/MODSA genetics. This is especially important for MODSA isolates, as they lack a phenotypic approach for their identification. Although we only detected two BORSA/MODSA isolates, it is important to note that all samples re-classified as MSSA presented cefazolin high inoculum effect (CzIE) (Table 1). This result must be highlighted as CzIE has been associated with therapeutic failure in invasive MSSA infections [8]. As it is known, cefazolin is a first-generation cephalosporin used as the preferred antibiotic treatment to combat MSSA infections. These isolates come from urine and blood cultures, demonstrating the high risk of therapeutic failure in MSSA infections when the bacterium has been established and disseminated.

Currently, the best way to identify BORSA/MODSA isolates is through a combined diagnostic approach that considers both molecular (e.g., *mecA* detection) and phenotypic methods (e.g., oxacillin susceptibility). However, the cefazolin inoculum effect (CzIE) in isolates lacking *mec* genes could be a helpful indicator for BORSA isolates, as both mechanisms are mediated by β-lactamase activity. Nonetheless, the limited BORSA samples in our study did not allow us to establish this correlation. Finally, defining which polymorphisms are related to β-lactams resistance in the previously mentioned determinants (e.g., PBPs, GdpP, AcrB, etc.) could be the best molecular approach for detecting these strains.

Molecular and phenotypic typing results demonstrate the complexity of monitoring *S. aureus* infection. As expected, MRSA isolates were the most prevalent; however, identifying isolates with barely described antibiotic resistance, such as BORSA/MODSA, underlines the importance of a combined diagnosis method to track *S. aureus*. Other studies have reported that only 0.1% of the isolates analyzed presented a BORSA phenotype, with a study timeframe between 2009 and 2013 [35]. This study confirms the presence of two isolates (3.5%) with such discordant profiles collected between 2019 and 2020. These results stand out for the potential increase in these difficult-to-identify profiles, with an increase of up to six times in 2016 [35]. Therefore, it is necessary to describe the resistance mechanism underlying these profiles and the clinical implications of their misidentification. Consequently, we emphasize the necessity of conducting a genomic analysis of these BORSA/MODSA isolates at a Latin American level to determine which genetic determinants are sufficient and necessary for β-lactam resistance in the absence of *mec* genes, as well as the virulence and antibiotic resistance molecular markers that can help us to establish epidemiological characteristics of isolates for healthcare and community environments.

## 5. Conclusions

*S. aureus* is one of the most important pathogens worldwide, not only because of its different clinical presentations, but also due to its high rate of resistance to many antibiotics with different mechanisms of action. Therefore, we evaluated the antibiotic sensitivity of a group of clinical *S. aureus* isolates, described their molecular and phenotypic traits, and found two isolates with different sensitivity patterns to β-lactam antibiotics. Those isolates were classified as BORSA/MODSA, which are difficult to detect using conventional methods in clinical environments. These results emphasize the necessity of a deeper genome description in those isolates, the characterization of their β-lactam resistance mechanism, the virulence factors within, and their epidemiological impact.

## Figures and Tables

**Table 1:** Characterization of isolates with presumptive discordant susceptibility profiles. The table presents the OXA MIC values, the detection of the *mecA* and *blaZ* genes, the results of the rapid chromogenic nitrocefin test for CzIE determination, and the cefazolin MIC values for standard (CZ) and high-inoculum (CZ HI) susceptibility assays. The final column provides the isolates’ resistance classification based on their resistance to β-lactams and the observed high-inoculum cefazolin effect (CzIE). +: gene present, −: gene absent.

Isolates	OXA MIC (μg/mL)	*Spa*-Type	*mecA*	*blaZ*	Chromogenic Rapid Test (BlaZ, CzIE)	CZ MIC (µg/mL)	CZ HI MIC (µg/mL)	Classification
7	2	t037	+	+	Not done	Not done	Not done	MRSA
28	1	Unknown	−	+	Positive	1	64	MSSA (CzIE+)
34	0.5	t743	−	−	Positive	0.5	≥64	MSSA (CzIE+)
37	16	t122	−	+	Positive	8	≥64	BORSA/MODSA
38	16	t9987	−	−	Negative	8	16	BORSA/MODSA (CzIE+)
44	0.5	Unknown	−	+	Negative	0.5	≥64	MSSA (CzIE+)
1634	32	t002	+	+	Positive	16	Not done	MRSA

## Data Availability

The original contributions presented in this study are included in the article/Supplementary Materials. Further inquiries can be directed to the corresponding author.

## Supplementary Materials

The following supporting information can be downloaded at: https://www.mdpi.com/article/10.3390/microorganisms12122598/s1. Figure S1: Maximum likelihood phylogenetic tree; Figure S2: Minimum spanning tree of *spa*-Typing and pandemic clones of *S. aureus*; Figure S3: Colorimetric rapid test with nitrocefin and induction with ampicillin to detect extracellular β-lactamases and high inoculum cefazolin effect (CzIE). Table S1: List of primers used to clinical isolate molecular characterization and the PCR conditions; Table S2: Antibiotic susceptibility profiles of β-lactam antibiotics and molecular typing by PCR endpoint and *spa*-typing. References [36,37] are cited in the Supplementary Materials.
