# Supplementary material for: Discordant β-Lactam Susceptibility in Clinical Staphylococcus aureus Isolates: A Molecular and Phenotypical Exploration to Detect the BORSA/MODSA Isolates in Bogotá, Colombia
Source: Microorganisms. 2024 Dec 16;12(12):2598. doi: 10.3390/microorganisms12122598 (PMC11679903; doi:10.3390/microorganisms12122598)
Supplement: Supplementary file 1 [file microorganisms-12-02598-s001.zip › microorganisms-3346346-supplementary.pdf]

Discordant β-lactam Susceptibility in Clinical *Staphylococcus aureus* Isolates: A Molecular and Phenotypical Exploration to Detect the BORSA/MODSA Isolates in Bogotá, Colombia

Angie Lorena Fonseca-Fernández[a†], María Alejandra Mancera-García[a†], Aura Lucia Leal-Castro[b], Chad Leidy[c], Sandra Rincón[d], Lina P. Carvajal[d], Jinnethe Reyes[d], Adriana Marcela Celis Ramírez[a*]

[a]Grupo de Investigación Celular y Molecular de Microorganismos Patógenos, Department of Biological Science, Universidad de los Andes, Bogotá D.C., Colombia. [b]Grupo de Investigación en Enfermedades Infecciosas, Faculty of Medicine, Universidad Nacional de Colombia, Bogotá D.C., Colombia. [c]Biophysics Group, Department of Physics, Universidad de los Andes, Bogotá, Colombia. [d]Molecular Genetics and Antimicrobial Resistance Unit, Universidad El Bosque, Bogotá D.C., Colombia.

*** Correspondence:**
Corresponding author
acelis@uniandes.edu.co

Keywords: MRSA; BORSA/MODSA; *spa*-typing; *pvl*; CzIE; *Staphylococcus aureus.*

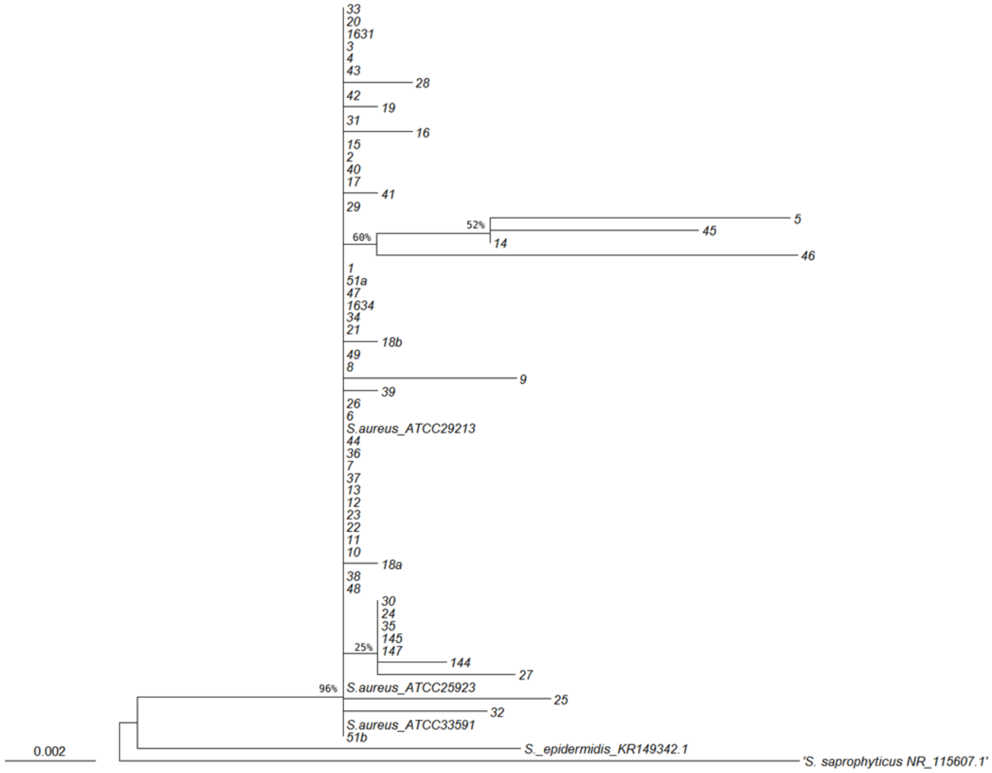

Figure S1. **Maximum likelihood phylogenetic tree.** (bootstrap:1000, evolutionary model: kimura 2) constructed with molecular marker 16s rRNA; as outgroups: *S. saprophyticcus* ATCC 15305

(NR_115607.1) and *S. epidermidis (KR_149342.1).* The evolutionary distance scale is at the lower part of the tree.

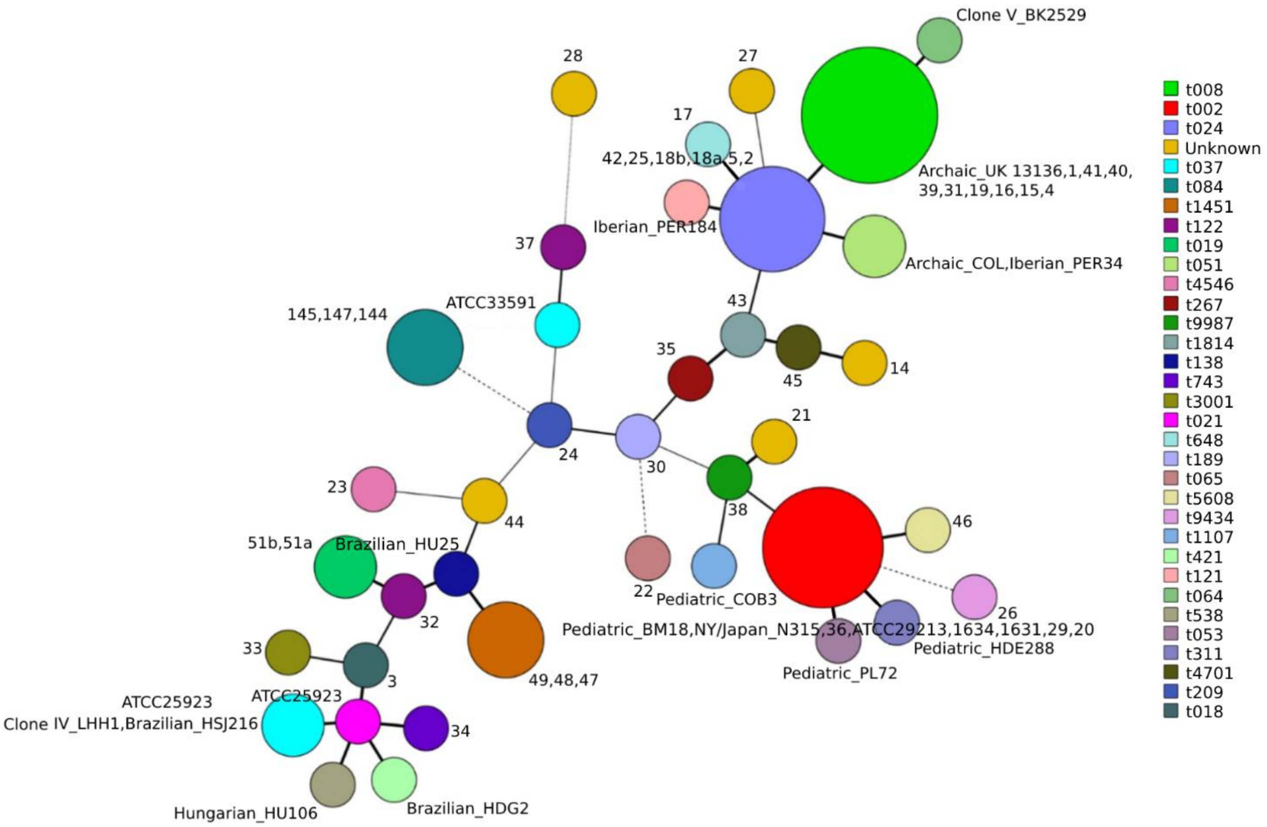

Figure S2. Minimum spanning tree of *spa*-typing and pandemic clones of *S. aureus* [24]. Colors indicate *spa*-type and line style the phylogenetic distance between nodes (thick solid line for differences up to 2.8; thin solid line for variations up to 4.59; thinner solid line for variations up to 6.4; dashed line for differences up to 8.19; and dotted line greater than 8.19).

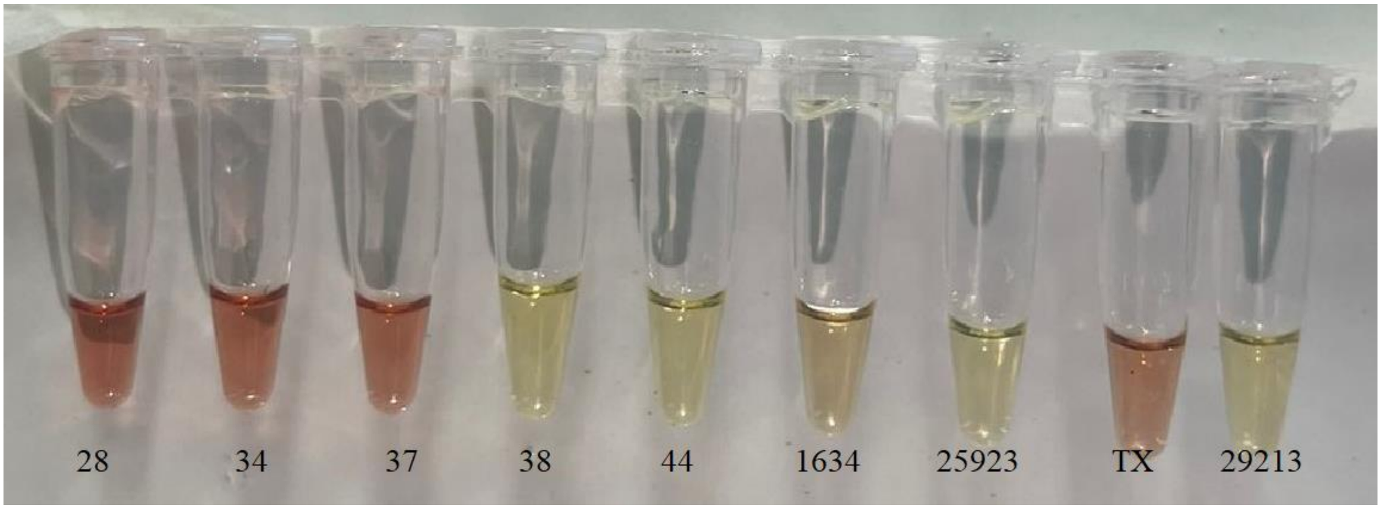

Figure S3. **Colorimetric rapid test with nitrocefin and induction with ampicillin to detect extracellular β-lactamases and high inoculum cefazolin effect (CzIE).** This assay was performed in the presumptive discordant antibiotic resistance isolates (28, 34, 37, 38, 34 and 1634). As quality controls we used the same already reported by Rincón and colleages 2021, TX0117 (BlaZ-positive exhibiting the CzIE), ATCC 29213 (BlaZ-positive lacking the CzIE), and ATCC 25923 (BlaZ-negative lacking the CzIE). A positive result to β-lactamases extracellular liberation and CzIE is considered by any colorimetric change from yellow to red in the tube sample.

Table S1. List of primers used to clinical isolates molecular characterization and the PCR conditions.

Gene	Primers	Amplicon size (pb)	PCR conditions
16S rARN<br>[36]	27F (5'-AGAGTTTGATCMTGGCTCAG)<br>1492R (5′-TACCTTGTTACGACTT)	1465	1 cycle of 3 min to 94 ° C<br>35 cycles of 45 seconds to 94 ° C,<br>45 seconds to 50 ° C and 45 second to 72 °,<br>1 cycle of 7 min a 72 ° C
mecA<br>[22]	*mecA*-F (5´-TGGTATGTGGAAGTTAGATTGGGAT)<br>*mecA*-R (5´-CTAATCTCATATGTGTTCCTGTATTGGC)	155	1 cycle of 5 min to 94 ° C<br>30 cycles of 1 min to 94 ° C, 1 min to 57 ° C,<br>2 min to 72 ° C and 5 min to 72 ° C<br>1 cycle of 5 min to 72 ° C
spa<br>[37]	Spa1095-F (5´-AAAGACGATCCTTCGGTGAGC)<br>Spa1517-R(5´-GCTTTTGCAATGTCATTTACTG)	Variable	1 cycle of 5 min to 94 ° C<br>30 cycles of 1 min to 94 ° C, 1 min to 55° C,<br>2 min to 72 ° C and 5 min to 72 ° C<br>1 cycle to 5 min to 72 ° C
pvl<br>[12]	Luk-PV-F(5´-ATCATTAGGTAAAATGTCTGGACATGATCCA)<br>Luk-PV-R (5´-GCATCAAGTGTATTGGATAGCAAAAGC)	433	1 cycle of 5 min to 94 ° C<br>30 cycles of 1 min to 94 ° C, 1 min to 60° C,<br>2 min to 72 ° C<br>1 cylce of 5 min to 72 ° C

Table S2. Antibiotic susceptibility profiles of β-lactam antibiotics and molecular typing by PCR end point and *spa - typing*.: OXA: oxacillin, FOX: cefoxitin and non-β-lactam antibiotics: GN: gentamicin, CIP: ciprofloxacin, E: erythromycin, DA: clindamycin and STX: trimethoprim-sulfamethoxazole. R: resistant, I: intermediate and S: susceptible. In the table are shown the absence (-) or presence (+) of *mecA* and *pvl* genes; finally, isolates *spa-types* are shown in the last column.

Isolate	β-lactam antibiotics		Non-β-lactam antibiotics					*mecA*	*pvl*	*spa*
	OXA	FOX	GN	CIP	E	DA	STX			
1	R	R	S	S	S	S	S	+	+	***t008***
2	R	R	S	S	S	S	S	+	+	**t024**
3	R	R	S	S	S	S	S	+	-	**t018**
4	R	R	S	S	S	I	S	+	+	**t008**
5	R	R	S	S	S	S	S	+	+	**t037**
6	R	R	I	S	S	S	S	+	+	**t008**
7	R	S	S	S	S	S	S	+	-	**t037**
8	R	R	S	S	I	S	S	+	+	**t008**
9	R	R	S	S	R	S	S	+	+	**t002**
10	R	R	S	S	S	S	S	+	+	**t648**
11	R	R	S	S	I	S	S	+	+	**t008**
12	R	R	S	S	R	S	S	+	+	**t002**
13	R	R	S	S	S	S	S	+	+	**t008**
14	R	R	S	S	R	S	S	+	+	**Unkown**
15	R	R	S	S	S	S	S	+	+	**t008**
16	R	R	S	S	S	S	S	+	+	**t008**
17	R	R	S	S	S	S	S	+	+	**t648**
19	R	R	S	S	S	S	S	+	+	**t008**
20	R	R	S	S	I	S	S	+	-	**t002**
21	S	S	S	S	S	S	S	-	-	**Unkown**
22	S	S	S	S	S	S	S	-	-	**t065**
23	S	S	S	S	S	S	S	-	+	**t4546**
24	S	S	S	S	S	S	S	-	+	**t209**
25	S	S	S	S	S	S	S	-	-	**t037**
26	S	S	S	S	S	S	S	-	-	**t9434**
27	S	S	S	S	S	S	S	-	-	**Unkown**
28	S	S	S	S	S	S	S	-	+	**Unkown**
29	S	S	S	S	I	S	S	-	+	**t002**
30	S	S	S	S	S	S	S	-	-	**t189**
31	S	S	S	S	S	S	S	-	+	**t008**
32	S	S	S	S	S	S	S	-	-	**t122**
33	S	S	S	S	S	S	S	-	+	**t3001**
34	S	S	S	S	S	S	S	-	-	**t743**
35	S	S	S	S	S	S	S	-	-	**t267**
36	S	S	S	S	I	S	S	-	-	**t002**
37	R	S	R	S	S	S	S	-	-	**t122**
38	R	S	S	S	R	R	S	-	-	**t9987**
39	R	R	S	S	S	S	S	+	+	**t008**
40	R	R	S	S	S	S	S	+	+	**t008**

**41**	R	R	S	S	S	S	S	+	+	**t008**
**42**	R	R	I	S	S	S	S	+	+	**t024**
**43**	R	R	S	S	S	R	S	+	-	**t1814**
**44**	S	S	S	S	S	S	S	-	-	**Unkown**
**45**	R	R	S	S	S	S	S	+	-	**t4701**
**46**	S	S	S	S	R	S	S	-	-	**t5608**
**47**	S	S	I	S	R	S	S	-	-	**t1451**
**48**	S	S	S	S	R	R	S	-	-	**t1451**
**49**	S	S	S	S	R	S	S	-	-	**t1451**
**144**	S	S	S	S	S	S	S	-	-	**t084**
**145**	S	S	S	S	S	S	S	-	-	**t084**
**147**	S	S	S	S	R	R	S	-	-	**t084**
**1631**	R	R	S	R	R	R	S	+	-	**t002**
**1634**	R	S	S	R	R	R	S	+	-	**t002**
**18a**	R	R	S	S	S	S	S	+	+	**t024**
**18b**	R	R	S	S	S	S	S	+	+	**t024**
**51a**	S	S	S	S	R	R	S	-	-	**t019**
**51b**	S	S	S	S	S	S	S	-	-	**t019**
